# Genome-Wide Analysis of LysM-Containing Gene Family in Wheat: Structural and Phylogenetic Analysis during Development and Defense

**DOI:** 10.3390/genes12010031

**Published:** 2020-12-29

**Authors:** Zheng Chen, Zijie Shen, Da Zhao, Lei Xu, Lijun Zhang, Quan Zou

**Affiliations:** 1 School of Applied Chemistry and Biological Technology, Shenzhen Polytechnic, 7098 Liuxian Street, Shenzhen 518055, China; chenzheng7@uestc.edu.cn (Z.C.); dazao5280@hotmail.com (D.Z.); c7zlj@szpt.edu.cn (L.Z.); 2 Institute of Fundamental and Frontier Sciences, University of Electronic Science and Technology of China, No.4 Block 2 North Jianshe Road, Chengdu 610054, China; shenzijie2013@163.com; 3 School of Electronic and Communication Engineering, Shenzhen Polytechnic, Shenzhen 518055, China

**Keywords:** LysM family, plant disease resistance, phylogenetic analysis, gene duplication, synteny, wheat

## Abstract

The lysin motif (LysM) family comprise a number of defense proteins that play important roles in plant immunity. The LysM family includes LysM-containing receptor-like proteins (LYP) and LysM-containing receptor-like kinase (LYK). LysM generally recognizes the chitin and peptidoglycan derived from bacteria and fungi. Approximately 4000 proteins with the lysin motif (Pfam PF01476) are found in prokaryotes and eukaryotes. Our study identified 57 LysM genes and 60 LysM proteins in wheat and renamed these genes and proteins based on chromosome distribution. According to the phylogenetic and gene structure of intron–exon distribution analysis, the 60 LysM proteins were classified into seven groups. Gene duplication events had occurred among the LysM family members during the evolution process, resulting in an increase in the LysM gene family. Synteny analysis suggested the characteristics of evolution of the LysM family in wheat and other species. Systematic analysis of these species provided a foundation of LysM genes in crop defense. A comprehensive analysis of the expression and cis-elements of LysM gene family members suggested that they play an essential role in defending against plant pathogens. The present study provides an overview of the LysM family in the wheat genome as well as information on systematic, phylogenetic, gene duplication, and intron–exon distribution analyses that will be helpful for future functional analysis of this important protein family, especially in Gramineae species.

## 1. Introduction

**Figure 1:**
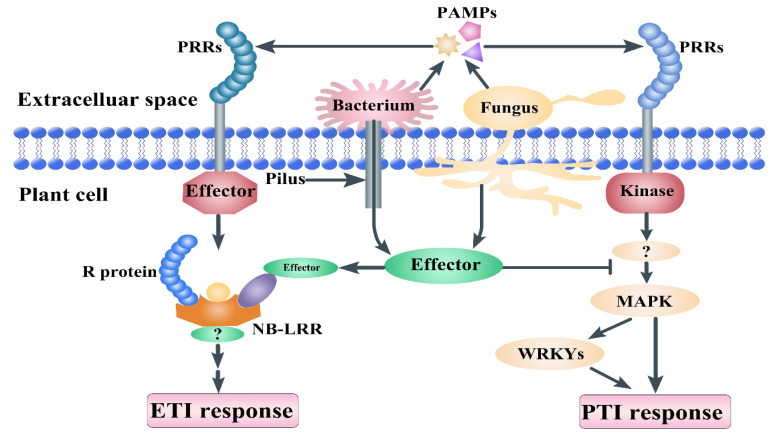
Plant immunity principles. Pathogen-associated molecular patterns (PAMPs) are molecules released from pathogens, such as chitin and flagellin. Pattern recognition receptors (PRRs) can recognize the PAMPs from the pathogens and transmit signals to mitogen-activated protein kinases (MAPKs) and WRKY transcript factors, which induce PAMP-triggered immunity (PTI) response. Pilus is a tool that delivers bacterial effector proteins into the host cell. PRRs consist of the leucine-rich repeat (LRR) domain and are recognized by nucleotide-binding site (NB)-LRR receptors, which induce effector-triggered immunity (ETI). NB-LRR proteins consist of an LRR domain, a NB domain, and an amino-terminal Toll/interleukin-1 receptor resistance protein (TIR) or a coiled-coil (CC) domain.

During the evolution process, plants and pathogens formed a complex and close relationship, with the most important relationship being infection and defense. Microbe-associated molecular patterns (MAMPs) are cell envelope components that include fungal chitin, bacterial lipopolysaccharide (LPS), bacterial flagellin, fungal chitin, and peptidoglycan (PGN) [1,2]. Lysin motif (LysM)-containing proteins are pattern recognition receptors (PPRs) that recognize MAMPs [3,4,5,6]. In plants, most defense proteins contain a typical LysM domain [7]. The identification of pathogens is an important part of the plant immune system. Many gene families take part in various stresses, such as disease resistance [8,9,10]. There is a recognition receptor containing a LysM domain on the membrane that binds to chitin on fungal cell walls and transmits the signal to activate immune response. The first line of inducible defense in plants is innate immunity. There are two general strategies involved in the detection of pathogens in plants [3,4,11]. Special receptors (PRRs) on the external membrane of the host cell recognize pathogen-associated molecular patterns (PAMPs) [12]. Plants also respond to cell wall or cuticular fragments that are released during pathogen invasion. PAMP-triggered immunity (PTI) occurs in response to the stimulation of PRRs. Effector-triggered immunity (ETI) is induced by recognizing signals from the receptors of pathogen molecules [13]. Previous studies have found that the receptor kinase BAK1 bound to multiple PRRs to mediate the activation of plant PTI (Figure 1). Flagellin proteins are derived from *Pseudomonas syringae* pv. *tabaci* 6605 and *flg22Pa* (QRLSTGSRINSAKDDAAGLQIA). The flg22 polypeptide is a highly conserved region at the N-terminal of bacterial flagellin, which can induce immune response in plants [13,14]. Chitin, a long-chain polymer of N-acetylglucosamine, is one of the primary components of cell walls in fungi [15]. The LysM family includes LysM-containing receptor-like proteins (LYP) and LysM-containing receptor-like kinase (LYK), which widely exist in the plant kingdom [16,17]. These genes take part in the recognition of chitin, lipopolysaccharide, and peptidoglycan, as well as MAMPs or PAMPs, and trigger plant immunity and legume root nodule formation. LysM consists of 44 amino acids and a repeat of seven amino acids at the C-terminal of the protein, and it was first found in the *Bacillus phage Φ29* lysozyme [18]. LysM was subsequently found in an enzyme named peptidoglycan hydrolase from the bacteria *Enterococcus faecalis* [19,20]. LysM is generally present in the degradation enzymes of bacteria and participates in degradation of the bacterial cell wall [6,21,22,23,24,25,26]. With the development of transcriptome and genome sequences, LysM domain proteins have been verified recently as widely existing in organisms [27,28]. 

OsCEBiP is the first receptor protein to contain two extracellular LysM domains and one transmembrane LysM domain that recognize PAMP chitin directly in rice. Silencing of *OsCEBiP* in transgenic plants almost completely abolishes their response to chitin [7]. *Arabidopsis* LYM1/LYM3 is sensitive to PGN rather than chitin in *Arabidopsis* [29]. OsLYP6 and OsLYP4 form a dimer that interacts with CEBiP and plays a complex role in PGN and chitin recognition [30]. In *Arabidopsis*, LYM1 and LYM3, as the LYPs, are plasma membrane proteins similar to the CEBiP protein and play an important role in the physical binding to PGNs [29]. Orthologs of OsCEBiP also appear to be involved in defense against pathogens. Knockdown of TaCEBiP showed disease symptoms in wild-type (WT) wheat plants [31]. A small RNA (Fg-sRNA1) from *Fusarium graminearum* could suppress wheat defense response by targeting and silencing a resistance-related gene that codes a chitin elicitor binding protein (TaCEBiP) [32]. OsCERK1 is a LysM receptor-like protein kinase that contains three lysin motifs and one kinase domain, and it is an essential factor for chitin signal transduction in rice [33]. As a plasma membrane protein, OsCERK1 does not interact with chitin directly but interacts with the chitin receptor OsCEBiP, which lacks a kinase domain [34,35], to transmit the chitin signal sensed by OsCEBiP to the cell. OsCERK1 is a critical component in basal resistance for plant innate immunity in the chitin signaling pathway [36]. A lack of AtLYK3 function affects the defense response signaling pathways regulated by ABA (abscisic acid) [37]. AtLYK4 has the same function in chitin signaling, and a mutation in AtLYK4 was found to decrease the induction of CRGs (chitin-responsive genes) and [Ca^2+^]cyt levels and enhance resistance to fungal and bacterial pathogens [38,39]. After chitin treatment, AtLYK5 leaves the plasma membrane via mobile intracellular vesicles, and AtCERK1 remains in the plasma membrane. Chitin-induced phosphorylation of AtCERK1 triggers the internalization of AtLYK5 [40]. AtLYK5 mediates the association between LYK4 and AtLYK1 [41]. NFR1 and NRF5 are two nod factor receptors in legumes that are also composed of LysM domains [42]. For chitin perception and immunity to fungal infection, the LYM1, LYM3, and AtCERK1 perception system in *Arabidopsis* is similar to the OsCEBiP-OsCERK1 complex [29].

Wheat, with three subgenomes, is a typical hexaploidy plant (AABBDD; *Triticum aestivum* L.), which underwent two separate allopolyploidization events during its formation. The first event occurred approximately 0.3 to 0.5 million years ago, in which the AA genome from *Triticum urartu* crossed with the BB genome from an unidentified species to produce the *Triticum turgidum* (AABB) species, which was a tetraploid wheat [43,44,45]. Approximately 10,000 years ago, the tetraploid wheat of *Triticum turgidum* hybridized with another DD genome plant species, named *Aegilops tauschii*, to produce the food plant hexaploid wheat (AABBDD) [44,46,47].

The present study analyzed LysM family genes in wheat. The entire protein sequence of LysM (pf01476) and the pkinase (pf00069) conservative domain was used as the seed sequence. Members of the LYP and LYK family in wheat were compared and analyzed in the entire genome range. The evolutionary relationship, chromosome distribution, and gene structure of their family members were analyzed. Based on the published transcriptome data, expression of the LYP and LYK family in wheat tissue, the development process, and biological stress were analyzed. Based on the role of the LysM domain in plant disease resistance, this study discusses the significance and trends of related research and provides a theoretical basis for the use of LysM domains for effective disease-resistance breeding.

## 2. Material and Method

### 2.1. Sequence Identification and Annotation of LYP and LYK Genes

The hidden Markov model (HMM) file of two conservative domains—LysM domain (PF01476) and kinase domain (PF00069)—were downloaded from the protein family database (Pfam) (http://pfam.sanger.ac.uk/) website. Wheat genome (Triticum_aestivum.IWGSC.dna.toplevel.fa, 2019), GFF3 file (Triticum_aestivum.IWGSC.45.gff3, 2019), and protein sequences (Triticum_aestivum.IWGSC.pep.all.fa, 2019) were downloaded from the ensembl plant website (http://plants.ensembl.org/index.html). 

All the genome information was downloaded from the ensembl website. The genome version of *T. dicoccoides*, *T. turgidum*, *H. vulgare,* and *O. sativa* were Triticum_dicoccoides.WEWSeq_v.1.0.dna.toplevel.fa, Triticum_turgidum.Svevo.v1.dna.toplevel.fa, Hordeum_vulgare.IBSC_v2.dna.toplevel.fa, and Oryza_sativa.IRGSP-1.0.dna.toplevel.fa, respectively. The gff3 file of the four species were Triticum_dicoccoides.WEWSeq_v.1.0.45.gff3, Triticum_turgidum.Svevo.v1.45.gff3, Hordeum_vulgare.IBSC_v2.45.gff3, and Oryza_sativa.IRGSP-1.0.45.GFF3, respectively, while the protein sequence file of the four species were Triticum_dicoccoides.WEWSeq_v.1.0.pep.all.fa, Triticum_turgidum.Svevo.v1.pep.all.fa, Hordeum_vulgare.IBSC_v2.pep.all.fa, and Oryza_sativa.IRGSP-1.0. pep.all.fa, respectively.

HMMER 3.0 (http://hmmer.org/download.html) was used to search for the LYP and LYK genes from the database, and the e-value was 1e-10 [48]. Clustalw (version 2.1) was used for multisequence alignment to build a new hidden Markov model file, which was used for sequence extracting. A cutoff value (0.01) was used for filtered sequences and deleted duplicates for these sequences. After identification of the LYP and LYK genes using HMMER, all of the candidate genes were examined using Pfam and NCBI (National Center for Biotechnology Information) to verify the core LysM sequences and the kinase domain of LYKs. Finally, the LYP and LYK genes were identified after comprehensive curation of the wheat genome. Sequences of the LYP and LYK genes, molecular weights, isoelectric points, length of sequences, GRAVY, and the subcellular location of identified LYPs and LYKs were predicted using the ExPasy website (https://www.expasy.org/). Together, the subcellular localization predictions of the LYP and LYK members were made using the website Plant-mPLoc (http://www.csbio.sjtu.edu.cn/bioinf/plant-multi/) [49]. 

### 2.2. Cis-Element and Expression Analysis of LYP and LYK Genes

The promoter sequences upstream of the LYP and LYK gene members were extracted from the wheat genome. Then, the cis-element was predicted from the SOGO website (https://sogo.dna.affrc.go.jp/cgi-bin/sogo.cgi?lang=en) using these sequences. Cis-elements related to plant resistance were chosen for our analysis. The expression data were adopted from the wheat expression website (http://www.wheat-expression.com/).

### 2.3. Phylogenetic Analysis and Classification of LYP and LYK Genes

MAFFT (version 7) was used for LysM-containing protein sequence alignment for rice, wheat, *Arabidopsis*, and barley protein sequences with the E-INS-i algorithm [50,51]. The parameters of MAFFT software were Gap opening penalty, 1.53; offset value, 0.0; and scoring matrix for amino acid sequences, BLOSUM62. Using the collected protein alignments from MAFFT (E-INS-i algorithm), IQ-TREE software (multicore version 1.6.12) was used to construct the phylogenetic tree (Nguyen et al., 2014). The best-fit model of the trees was JTT+R4 [52]. Similar to the Shimodaira–Hasegawa method, phylogenetic assessment was assessed using ultrafast bootstraps, and 1000 replicates each were used [53,54,55,56,57]. The tree file was visualized by FigTree v1.4.3 (http://tree.bio.ed.ac.uk/software/figtree/).

### 2.4. Chromosomal Distribution and Gene Structure Analysis for LYP and LYK Genes

The gff3 file downloaded from Ensembl website was used in this step. To analyze the transcript structure, gtf file type was adopted, which was converted from gff3 file type using the gffread command in the cufflinks package. Then, LYK and LYP transcript structure data were extracted from the gtf file. MEME (http://meme.nbcr.net/meme/intro.html) [58] analysis of the LYP and LYK genes motif locations set the parameters as motif length from 6 to 50 residues with a maximum of 10 motifs and maximum size of 60,000. Online tools of the Gene Structure Display Server (GSDS: http://gsds.cbi.pku.edu.cn) [59] was used for visualization of the exon–intron distribution and motif location of LYP and LYK genes in wheat with the necessary sequence analyses in the results. The MapGene2Chrom website (http://mg2c.iask.in/mg2c_v2.1/) was used to visualize chromosomal distribution analysis [60]. 

### 2.5. Gene Duplication and Synteny Analysis

The function blastall of the BLAST software (version 2.2.26) was used to choose the duplicated LYP and LYK genes with e-value 1e-20. The Shinycircos software [61] was used to analyze gene duplication and chromosome mapping. For the synteny analysis, we used the Multiple Collinearity Scan toolkit (MCScanX: https://github.com/tanghaibao/jcvi/wiki/MCscan-(Python-version)) to analyze the gene duplication events (Wang et al., 2012); the parameter was minspan = 20.

## 3. Results

### 3.1. Identification of LYPs and LYKs in Wheat

A total of 60 coding sequences corresponding to Pfam LYP were identified and renamed for further analysis in the wheat genome. There were 19 LYK members in the LysM domain protein family (Supplement Table S1). Among all the coding sequences, two genes with three splice variants were kept in the dataset from each genomic locus (Table S1). LYK members have an extra kinase domain that is not there in LYP.

For the analysis of gene characteristics, protein sequences, protein molecular weight (MW), theoretical pI (pI), grand average of hydropathicity (GRAVY) (http://web.expasy.org/protparam), and subcellular localization (http://www.csbio.sjtu.edu.cn/bioinf/plant-multi/) were predicted from the website (Table S1). Among the 60 LYPs and LYKs, the Ta4ALysM-RLK1 protein with 749 amino acids was the largest protein, and Ta3B-LysM2 with 100 amino acids was the smallest protein. The ranges of MW, PI, and GRAVY were 10.3 (Ta3A-LysM6) to 80.5 kDa (Ta4ALysM-RLK1), 4.57 (Ta7A-LysM1) to 9.29 (Ta4A-LysM1), and −0.636 (Ta7D-LysM2) to 0.579 (Ta3D-LysM1), respectively. Many LYPs have been verified as cell membrane proteins, such as AtLYK4, AtLYK5, AtCERK1, OsCEBiP, and OsCERK1 [7,33,35,38,40,62,63]. Therefore, the prediction of subcellular localization results showed 41 cell membrane proteins, 16 chloroplast proteins, 16 nucleus proteins, and 3 cytoplasm proteins; the ratio of cell membrane LYP was greater than 68% for all of the LysM domain protein members.

### 3.2. Phylogenetic Tree Construction and Intron–Exon Distribution Analyses of LYP and LYK Genes

**Figure 2:**
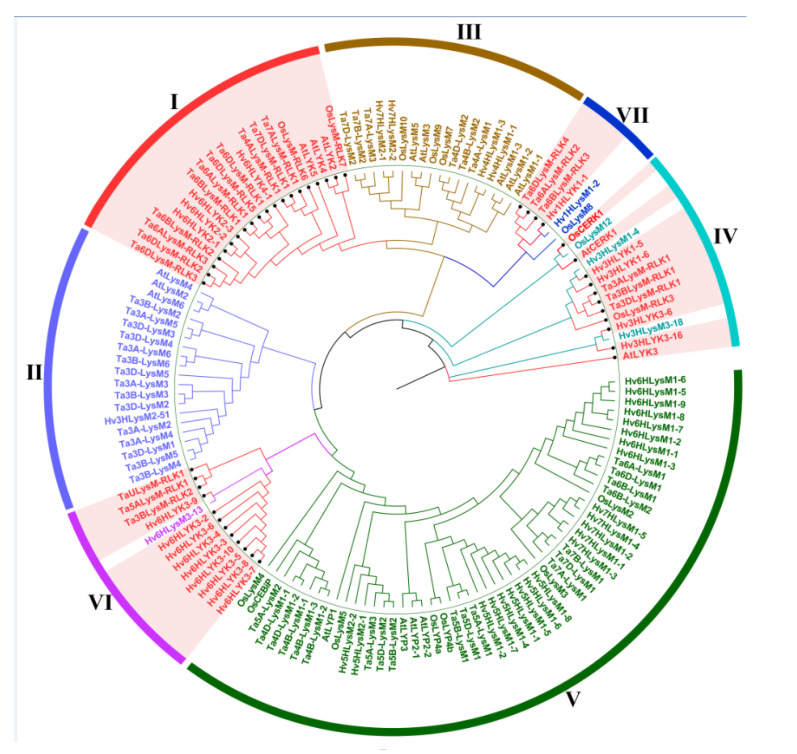
Phylogenetic tree of lysin motif (LysM)-containing receptor-like protein (LYP) and LysM-containing receptor-like kinase (LYK) in wheat, barley, *Arabidopsis*, and rice. Different colored arcs represent different groups of LYP and LYK. The red colored genes are LYKs distributed in different groups. LYK and LYP from *Arabidopsis*, barley, wheat, and rice are labeled with the prefix ‘At’, ‘Hv’, ‘Ta’, and ‘Os’, respectively.

To better understand the phylogenetic relationship of LYPs and LYKs in wheat and other plants, approximately 142 LYP and LYK sequences were used to analyze the intron–exon distribution and construct a phylogenetic tree (Figure 2). The tree consisted of 60 sequences primarily from *Triticum aestivum* L., 16 sequences from rice (*Oryza sativa* L.), 17 sequences from *Arabidopsis thaliana* L. and barley (*Hordeum vulgare* L.), and 49 protein sequences. 

The results indicated that the 142 LYP and LYK sequences could be divided into seven groups according to the intron–exon distribution and the phylogenetic relationship for wheat. Four groups primarily consisted of LYKs. For example, all of the proteins in group I were LYK. Group VI contained 12 LYKs among its 13 members (along with one LYP (Hv6HLysM3-13)), whereas there were 10 and 4 LYKs in group IV and group VII, respectively.

**Figure 3:**
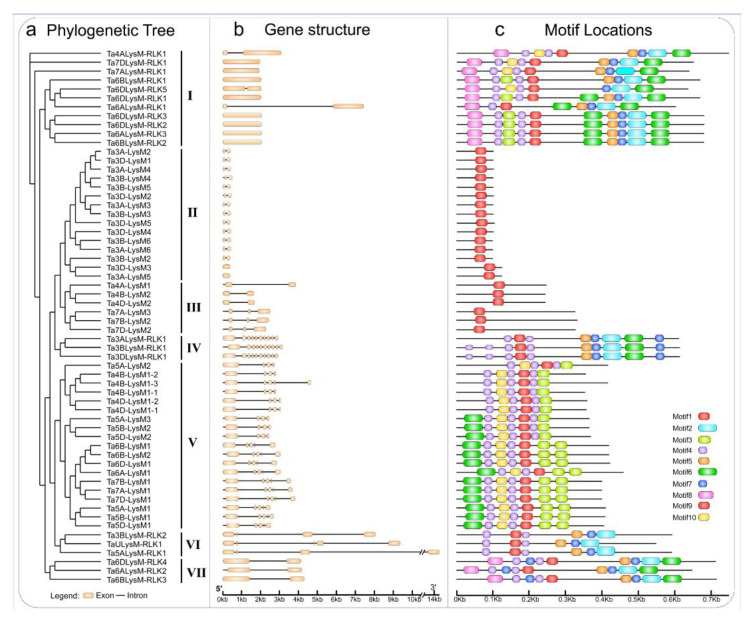
Phylogenetic tree, motif locations, and gene structures of LYPs and LYKs in wheat. (**a**) The phylogenetic tree of LYPs and LYKs in wheat. The LYPs and LYKs are divided into seven groups; group I, group IV, group VI, and group VII are the LYKs. (**b**) Gene structure of LYP and LYK genes. Black lines indicate introns, yellow boxes indicate exons, and the genomic length is indicated at the bottom. (**c**) Motif composition of LYPs and LYKs. Different colored boxes indicate motifs 1–10, and the information for each motif is in Supplementary File 1. The scale of the length of proteins is at the bottom.

The gene structures of the intron–exon distribution of all 60 LYPs and LYKs were investigated to further validate the evolution and phylogenetic relationships of LYP and LYK family members in wheat (Figure 3). The LYK genes in the same group in the phylogenetic tree shared similar structure of intron–exon organization.

### 3.3. Motif Composition of Wheat LYP and LYK Gene Family

The MEME motif analysis tool was used for the detection of protein sequences in wheat. As shown in Figure 3, motif 1, a LysM domain, was widely distributed in all of the LYPs and LYKs. Motif 2, a Pkinase domain, was unique to LYKs in groups I, III, VI, and VII. As shown in Figure 3c, the LYP and LYK member motif compositions were generally similar within the same group. For example, motif 7 and motif 5 existed in LYKs. Motif 1 was present in the LYP and LYK members of all groups. The clustered LysM protein groups, i.e., groups II, III, VI, and VII, showed highly similar motif distributions within the group. Overall, analysis of the conserved motif compositions and gene structures of LysM family genes and the phylogenetic analysis confirmed the group classifications in wheat.

### 3.4. Chromosomal Distribution of Wheat LysM and LysM-RLKs Genes

**Figure 4:**
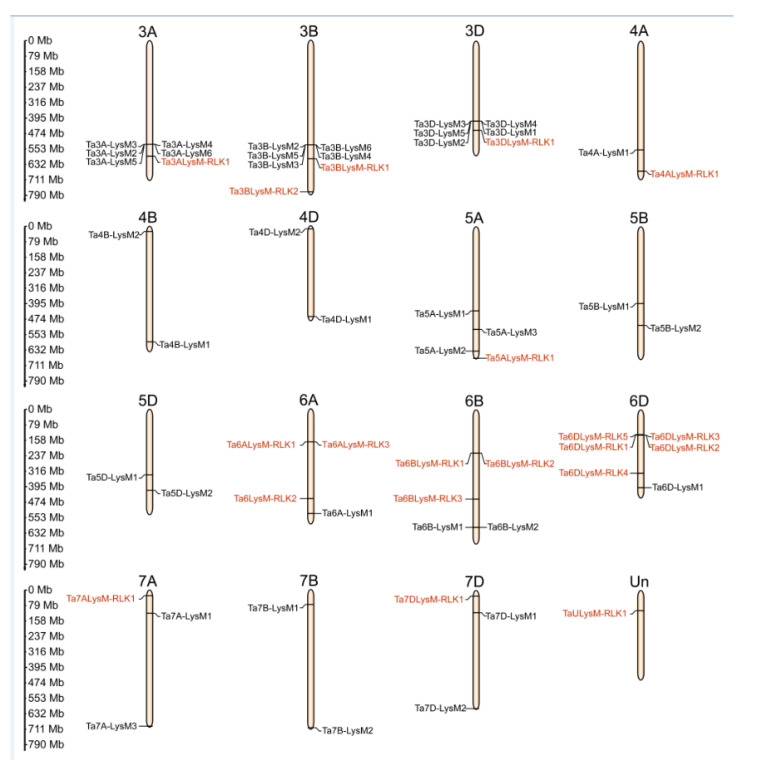
The chromosomal distribution of LYP and LYK genes in wheat. Distribution of LYP genes (black) and LYK genes (red) in the three subgenomes. The numbers at the left represent the length of the chromosomes and also provide the locations of these genes on their chromosomes.

The hexaploidy wheat genome comprises 42 chromosomes that range from 830.8 (chr3B) to 473.6 (chr6D) Mb. The LYP and LYK members are localized at chr3A, chr3B, chr3D, chr4A, chr4B, chr4D, chr5A, chr5B, chr5D, chr6A, chr6B, chr6D, chr7A, chr7B, chr7D, and Un (sequence region unknown). The chromosomal localization of genes encoding LYPs and LYKs is shown in Figure 4. Analysis of the chromosome distribution results showed that approximately 19 LYKs and 38 LYPs exhibited an uneven distribution in the 42 chromosomes. There were 5 LYKs at chr6D, 3 LYKs at chr6B, 2 LYKs at chr3B and chr6A, and only 1 LYK in the remaining chromosomes, such as chr3A, chr3D, chr4A, chr5A, chr7A, chr7D, and chrUn. Therefore, the number of LYKs were 6, 5, and 7 in subgenomes A, B, and D, respectively. However, there were no LYP or LYK members on chr1A, chr1B, chr1D, chr2A, chr2B, or chr2D. Genes belonging to a gene family were often distributed in clusters, such as 7 LYPs in chr3B and 6 LYPs in chr3A, chr3D, and chr6D. Among the subgenomes 3A, 3B, and 3D, the chromosome of 3B contained a Ta3BLys-RLK2 without a homologous in 3A and 3D. Chromosome 7B lacked a LYP gene in 7A and 7D, while 4A contained only one LYK gene among the subgenomes 4A, 4B, and 4D. There were two more genes in the 5A chromosome with one LYK gene compared to 5B and 5D. An enormous amount of LYK genes and huge divergence appeared among the subgenomes 6A, 6B, and 6D.

### 3.5. Wheat LYP and LYK Genes Show a High Rate of Gene Duplications

**Figure 5:**
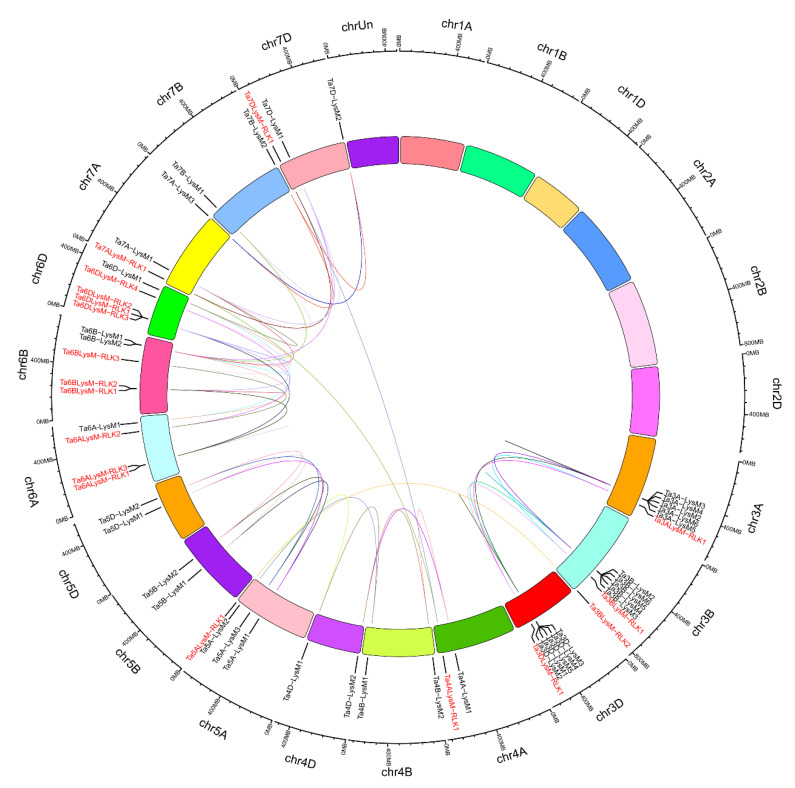
The interchromosomal relationships of LYP and LYK genes in wheat. The colored lines inside indicate duplicated LYP and LYK gene pairs. Distribution of LYP genes (black) and LYK genes (red) in the three subgenomes. The chromosome number is shown on the outside of the chromosomes. The numbers at the outside represent the length of the chromosomes and also provide the locations of these genes on their chromosomes.

To investigate the gene duplication of LYP and LYK genes in wheat, the LYP and LYK gene members were used for the analysis. The results suggested that LYPs and LYKs were unevenly distributed within 15 wheat linkage groups (LGs) (Figure 5). LG 3B contained the largest number of gene members with 6 LYPs and 1 LYK, while there were 6 genes in LG 3A and LG 3C. However, some linkage groups (e.g., LG 7A and LG 7D) had only one gene, and there were no genes in the other LGs, such as LG 1A, 1B, 1C 2A, 2B, and 2C. The chromosomal distribution and gene duplication had no positive correlation with the number of LysM family members and the LG length. 

According to previous research, a tandem duplication event is defined when there are two or more genes inside 200 kb [64]. Figure 5 shows that 16 LYM and LYP genes (*Ta3A-LysM3*/*Ta3A-LysM4*/*Ta3A-LysM2, Ta3A-LysM6*/*Ta3A-LysM5, Ta3B-LysM2*/*Ta3B-LysM6, Ta3B-LysM4*/*Ta3B-LysM3, Ta3D-LysM3*/*Ta3D-LysM4*/*Ta3D-LysM5, Ta3D-LysM1*/*Ta3D-LysM2, and Ta6DLysM-RLK3*/*Ta6DLysM-RLK1*) were classified into seven event regions—3A, 3B, 3D, and 6D—in wheat. Tandem duplication events were concentrated on chromosomes 3A, 3B, and 3D because two clusters were found in each. LG 6D had only one cluster of LYK genes. Therefore, the LYK and LYP genes likely occurred via gene duplication events during the evolutionary process.

### 3.6. Synteny Analysis for LYKs and LYPs in Wheat and Other Species

**Figure 6:**
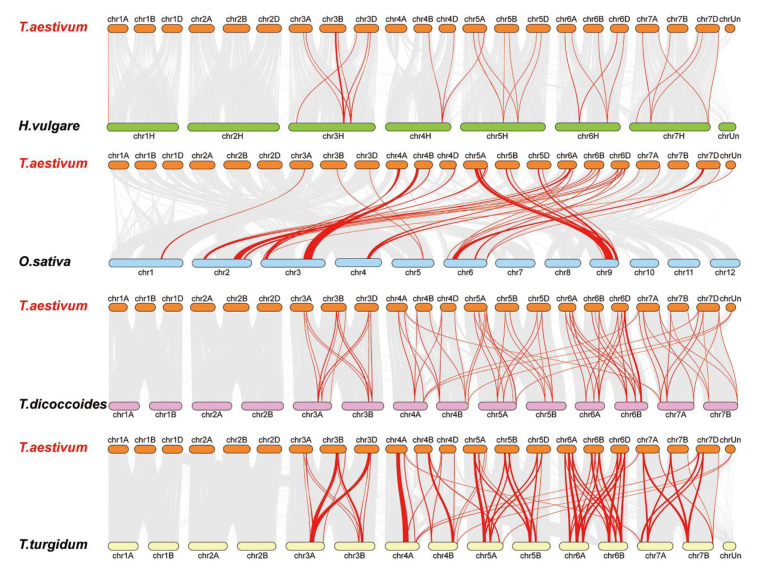
Synteny analysis of the LysM family. Red lines are the syntenic LYP and LYK gene pairs for different species. Gray lines are collinear blocks for plant genomes. The species are *Triticum aestivum*, *Hordeum vulgare*, *Oryza sativa*, *Triticum dicoccoides*, and *Triticum turgidum*.

Four comparative syntenic maps were constructed to verify the phylogenetic mechanisms of the LysM family in wheat using four species: one genus *Oryza* plant (*Oryza sativa*), one genus *Hordeum* plant (*Hordeum vulgare*), and two genus *Triticum* plants (*Triticum dicoccoides* and *Triticum turgidum*) (Figure 6). The results of the synteny analysis indicated that 38 genes in the wheat LysM family had a syntenic relationship between wheat and *T. dicoccoides*, *T. turgidum*, *H. vulgare*, and *O. sativa* (Table S2). The number of orthologous pairs of the LysM family in wheat and other species (*T. dicoccoides*, *T. turgidum*, *H. vulgare* and *O. sativa*) were 35, 34, 21, and 25, respectively. 

Some LysM family members were associated with more than one syntenic gene pairs between wheat and *T. turgidum*, for instance *Ta3D-LysM2*, *Ta3A-LysM4*, *Ta3A-LysM6*, *Ta3B-LysM4,* and *Ta3D-LysM5* were associated with *TRITD3Av1G201740.1* and *TRITD3Av1G202040.12*, while *Ta3B-LysM6* was associated with *TRITD3Bv1G178180.1* and *TRITD3Bv1G178570.1*. Significantly, Some LysM family members were associated with more than one syntenic gene pairs between wheat and *T. turgidum*. For example, *Ta3D-LysM2*, *Ta3A-LysM4*, *Ta3A-LysM6*, *Ta3B-LysM4,* and *Ta3D-LysM5* were associated with *TRITD3Av1G201740.1* and *TRITD3Av1G202040.12*, while *Ta3B-LysM6* was associated with *TRITD3Bv1G178180.1* and *TRITD3Bv1G178570.1*. Significantly, some LysM family syntenic gene pairs between wheat and *T. turgidum* were considered conserved syntenic blocks. Figure 6 shows that some syntenic gene pairs between wheat and *T. dicoccoides*/*H. vulgare*/*T. turgidum* were not found between wheat and rice, such as *Ta3BLysM-RLK1* and *HORVU3Hr1G084510.4*/*TRIDC3AG053320.1*/*TRITD3Av1G221050.1*, which suggests the formation of orthologous pairs after the divergence of evolution. Fourteen genes had a syntenic relationship between wheat and the other four species.

These results indicate that some orthologous gene pairs existed before ancestral divergence, and some divergence was generated among the different species during evolution. 

### 3.7. Analyses of Cis-Regulatory Element for LYKs and LYPs in Wheat

**Figure 7:**
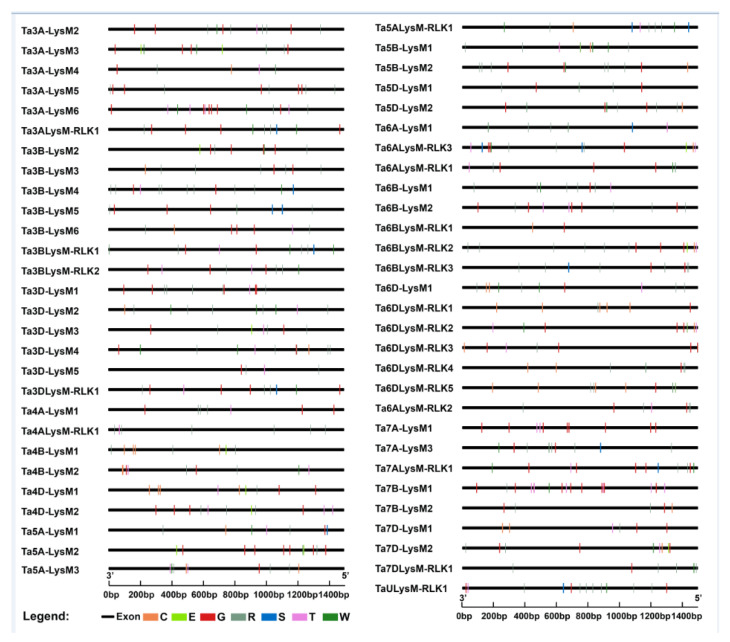
Cis-element analysis of the LysM gene family. Different colors and capital letters represent different cis-elements. G: GT1CONSENSUS, W: WBOXATNPR1, R: WRKY71OS, C: GCCCORE, T: GT1GMSCAM4, S: SEBFCONSSTPR10A, E: ELRECOREPCRP1.

To further investigate the regulatory mechanism of the LysM gene family members, the cis-regulatory elements were extracted in the promoter regions of LysMs (Table S3). A 1500 bp sequence upstream of the translational start site was considered as a putative promoter region and was thus used to analyze the distribution of cis-regulatory elements [65,66]. The cis-regulatory elements were indicated by capital letters labeled with different colors characterizing pathogen resistance, which implies that the LysM gene expression is associated with the stimulation responsiveness of pathogens. Seven cis-regulatory elements were detected for promotors of the LysM gene family members, such as GT1CONSENSUS (S000198), WBOXATNPR1 (S000390), ELRECOREPCRP1 (S000142), and so on (Figure 7). 

### 3.8. Analyses of Gene Expression of LYKs and LYPs in Response to Pathogens

**Figure 8:**
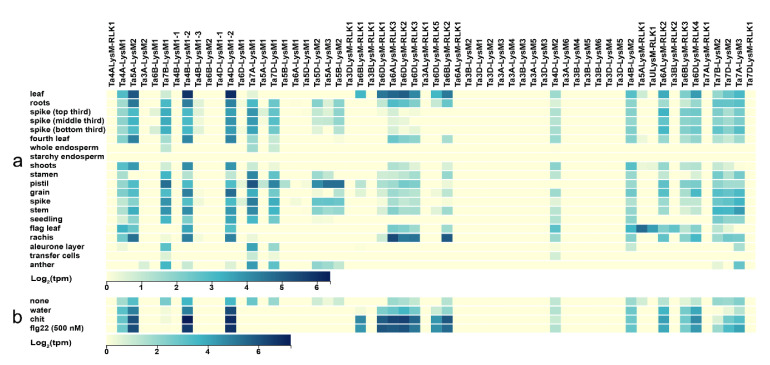
Expression of LysM genes in wheat. (**a**) Heat map of expression data in different Chinese spring tissues. (**b**) Heat map of expression data for different treatments. The blue color stands for high expression and the yellow color represents low expression. Water: water treatment; none: no treatment; chit: chitin; flg22: a polypeptide of flagellin proteins.

The expression data for the 57 LysM genes were adopted from the wheat expression website (http://www.wheat-expression.com/) [67,68]. The Chinese spring tissues of leaf, root, spike, shoots, stamen, pistil, spike, stem, flag leaf, rachis, anther, endosperm, grain, transfer cells, and the whole seedling were chosen for analysis of LysM gene members. The expression levels of the 60 LysM proteins in different tissues were used to construct a heat map (Figure 8a). LysM gene members were rarely expressed in the aleurone layer, transfer cells, and anther. *Ta4B-LysM1-2* and *Ta4D-LysM1-2* had the highest expression in leaves. Compared with the flag leaf, the leaf tissue had higher expression except for *Ta5ALysM-RLK1*, *TaULysM-RLK1,* and *Ta3BLysM-RLK2,* which were all LYKs belonging to group VI. These three genes had higher expression in the flag leaf, which might be related to grain development. LysM gene members *Ta7A-LysM1, Ta4D-LysM1-2, Ta6ALysM-RLK3,* and *Ta7A-LysM1* had the highest expression level in the Chinese spring tissues of pistil, grain, rachis, stamen, respectively. 

Flg22 and chitin act as elicitors that trigger the plant defense response. The expression data were taken from the wheat expression website. As shown in Figure 8b, no treatment and water treatment were used as control groups, and Chinese spring leaf was used as the experimental material. After treatment with chitin and FLG22, the LYP genes *Ta4A-LysM1*, *Ta5A-LysM2*, *Ta4B-LysM1-2*, and *Ta4D-LysM1-2* and the LYK genes *Ta6BLysM-RLK1*, *Ta6BLysM-RLK2*, *Ta6DLysM-RLK1*, *Ta6DLysM-RLK2*, *Ta6DLysM-RLK3*, *Ta6DLysM-RLK5*, and *Ta6ALysM-RLK3* were expressed higher than in the control group. Therefore, the LysM gene family had the function of increasing the resistance of wheat.

## 4. Discussion

The LysM domain is one of the most significant constructions in resistance genes in the immune system, and it is ubiquitous in the entire plant kingdom [36]. The LysM gene family has been identified and functionally characterized in many plants, and most of these genes are associated with plant immunity [29,35,40,69,70,71]. During the evolutionary process, the LysM gene family expanded via gene duplication [72,73,74,75], and the LysM gene family is large in plant species. Our study identified and analyzed 57 gene members in the wheat genome, which were renamed based on the chromosomal location. LysM proteins in different kingdoms have evolved specialized architectures and likely have distinct functions. For example, the LYKs of legume have been defined as the putative receptors of nodulation factors. However, in *Arabidopsis*, LYKs are found as chitin recognition proteins, which evolved from nodulation factor to recognition proteins. LYKs contain a kinase domain that does not exist in LYP. OsCEBiP plays a role in chitin elicitor binding, and OsCERK1 functions as a signal transducer through its Ser/Thr kinase activity in rice [35]. Therefore, the LysM genes that are expanding in wheat genomes have possibly acquired critical new functions in wheat. We assigned these gene members to seven groups. LYK genes existed in groups I, IV, VI, and VII, which was mutually supported by the exon–intron structure and phylogeny. 

The LysM genes were divided into LYP (without kinase domain) and LYK (with kinase domain) based on the intron–exon distribution of the gene structure and motif analysis. The LYK structure was also predicted with an N-terminal signal peptide, the LysM, transmembrane region, and kinase domain in the cytoplasmic part. The extracellular regions contained different numbers of LysM [33,76]. The LYPs were predicted to contain extracellular LysM and a transmembrane region without the kinase domain [7,77]. The chromosome distribution results showed no LysM genes located on chromosomes 1A, 1B, 1C, 2A, 2B, or 2C. Most of the LYK genes were distributed in chromosomes 6A, 6B, and 6D, making up approximately 52.6% of all the LYK genes in the wheat genome.

Tandem and gene duplications led to LysM gene expansion. There were only two genes—*Ta6DLysM-RLK5* and *TaULysM-RLK1*—that did not have cascade repeating genes. LysM genes in wheat have many tandem clusters, which reveals the contribution of the LysM gene family expansion. Genes that take part in development are generally rarely involved in gene duplication in rice and follow monocot–dicot separation, but defense or resistance genes, such as the LysM genes, participated in preferential expansion during the evolutionary process [78]. The large number of tandem and gene duplications for the LysM family in wheat reveal that these genes have important functions in plant innate immunity, such as the activity of LYK members against peptidoglycan and chitin treatment. 

For analysis of the cis-element, seven related to the resistance of plants were chosen. The GT1CONSENSUS (S000198) cis-element binds GT-1-like factors to the PR-1a promoter, influencing the level of salicylic acid (SA)-inducible gene expression [79,80,81]. As the "W-box" cis-element, WBOXATNPR1 (S000390) is explicitly recognized by SA-induced WRKY DNA binding proteins and acts as a harmful regulatory element for the inducible expression of *WRKY18* in *Arabidopsis* thaliana. SA usually participates in the resistance of plants [82,83,84,85,86]. As the core W-box, WRKY71OS (S000447) can bind W-box elements within *pathogenesis-related class 10* (*PR-10*) genes, which are the typical genes for plant resistance [83,87,88]. SEBFCONSSTPR10A (S000391) has been found in the pathogenesis-related gene (*PR-10a*) promoter and is similar to the auxin response element [89]. As the elicitor responsive element core of parsley *PR1* genes, ELRECOREPCRP1 (S000142) is bound by the WRKY1 protein [83,90,91]. As the core of GCC-box, GCCCORE (S000430) is found in many pathogen-responsive genes, such as *PDF1.2*, *Thi2.1*, and *PR4,* and appears to play important roles in regulating jasmonate-responsive gene expression [92,93]. GT1GMSCAM4 (S000453) plays a role in pathogen- and salt-induced gene expression [94]. Analyses of cis-regulatory elements have indicated that the promotor contains many cis-elements associated with plant resistance. These genes are probably related to disease resistance in wheat.

It is widely believed that bread wheat (*Triticum aestivum* L.; genome AABBDD) has undergone two sequential allopolyploidization events. First, allotetraploidization occurred between two diploid species—*T. urartu* (AA) and an unknown close relative of *Aegilops speltoides* (SS)—resulting in an extant tetraploidemmer wheat (*T. turgidum*; AABB) [44,46,47]. Second, allohexaploidization occurred between tetraploidemmer wheat and goat grass (*A. tauschii*; DD), leading to the formation of bread wheat [95]. There are 19 LysM genes in the A subgenome, 18 LysM genes in the B subgenome, and 19 LysM genes in the D subgenome among the 57 LysM genes in wheat, along with one LysM gene with an unknown chromosome. Although the total number of genes in the subgenomes is equal, the number of genes is different among homologous chromosomes. In wheat, gene replication also appears in other gene families, such as the MIKC-type MADS-box gene family. A previous study found the extensive expansion of some MIKC-type subfamilies, especially those potentially involved in adaptation to different environmental conditions, such as flowering-time genes. The topology of the MIKC-type MADS-box gene family is more complex, suggesting multiple duplication events before and/or after polyploidization of wheat [96]. Similarly, the duplication of LysM genes gained a balance status regarding the total number of genes in the subgenomes during wheat evolution. As a tetraploid species, *T. turgidum* (AABB) is the ancestor of bread wheat. A total of 34 LysM genes were found according to synteny analysis between wheat and *T. turgidum*, while 37 LysM genes were found according to synteny analysis between *T. dicoccoides* and *T. aestivum*. Probably gene gain has occurred during evolution from tetraploid *T. turgidum* and *T. dicoccoides* to hexaploid bread wheat, and the subgenome of goat grass (*A. tauschii*; DD) might have brought the new LysM genes. LysM genes were not the same in the three subgenomes. The synteny analysis results showed 21 LysM genes, indicating that gene loss occurred between wheat and barley. Sequence alignment of these synteny genes suggested that multiple SNPs loci existed. However, the similarity of the synteny sequences was approximately 95%. Gene sequences of different subgenomes were not identical, and there were multiple SNP loci between the duplicated genes with a high similarity. Synteny analysis of LysM genes in different plant species helped determine the potential functional roles and valuable clues to the evolutionary characterization of LysM genes and the genetic development of agronomic characteristics and plant resistance in wheat. 

To characterize the expression of wheat LysM genes, we analyzed RNA-seq data of 60 wheat LysM transcripts. Out of the 60 full-length transcripts, 57% (34 transcripts) were expressed in at least one developmental stage, 45% (27 transcripts) were expressed in more than two developmental stages, and there was at least one stage with a high expression level. The remaining 43% (26 transcripts) of full-length genes showed a deficient expression and were considered as not expressed. A total of 24 transcripts were expressed ubiquitously with a high expression level in the wheat plant. All of the LysM genes on chromosome 3 had no expression, except for Ta3A-LysM2 in the anther tissue and Ta3BLysM-RLK2 in the flag leaf. Most of the low-expression genes were focused on subfamily II and subfamily IV from the phylogenetic tree result. This analysis shows that genes from one subfamily can differ considerably in their expression pattern. The expression pattern was very similar to the MIKC-type MADS-box gene family in wheat [96]. There were no genes expressed in starchy endosperm cells and only three genes—*Ta7A-LysM1, Ta7B-LysM1,* and *Ta7B-LysM1*—were expressed in the whole endosperm tissue. These results indicate that LysM genes are not constitutively expressed.

The low-expression genes of each tissue in subfamily II and subfamily IV also had low expression or no expression in the flg22 group, chitin group, and the control group. There was a significant increase in high-expression genes in the flg22 group and chitin group compared with the water and no treatment groups, especially the LYK genes. These results indicate that LYKs play an important role in preventing the invasion of bacteria and fungus. At present, there has been little research focusing on the resistance function performed by the LysM family in wheat, so it is of great research significance to elucidate the molecular mechanism of resistance to pathogens. The prediction of LysM family members can provide materials for research on resistance in wheat. 

## 5. Conclusions

In our study, a comprehensive analysis of LysM gene family was performed in wheat. 57 gene members and 60 proteins were distributed all the genome expect chromosome 1 and chromosome 2. Based on the exon-intron structures and motif compositions, the proteins characterized and further classified into seven main groups. Synteny analysis and phylogenetic comparison of LysM genes from several different plant species provided valuable clues about the evolutionary characteristics of the LysM genes. The expression data suggested that LysM genes played important roles in wheat resistance of pathogens during growth and development. The phylogenetic and gene expression analysis will shed light on the functional analysis of LysM genes. These results provide a valuable resource for better understanding the biological roles of individual LysM genes in wheat.

## Data Availability

Data is contained within the article or supplementary material.

## Supplementary Materials

The following are available online at https://www.mdpi.com/2073-4425/12/1/31/s1, File S1: Details of the conserved motif in LysM family members. Table S1: Details of the LysM family proteins in wheat. Table S2 one-to-one orthologous relationships. Table S3 Details of the cis-element for LysM family in wheat.
