# Supplementary material for: Genome-Wide Analysis of LysM-Containing Gene Family in Wheat: Structural and Phylogenetic Analysis during Development and Defense
Source: Genes (Basel). 2020 Dec 29;12(1):31. doi: 10.3390/genes12010031 (PMC7823900; doi:10.3390/genes12010031)
Supplement: Supplementary file 1 [file genes-12-00031-s001.zip › Supplement file 1 Details of the conserved motif in LysM family members.doc]

Supplement file 1 Details of the conserved motif in LysM family members.
